# Design of fault water-resisting coal pillars based on deep-beam analysis: A comparison of two analytical methods

**DOI:** 10.1371/journal.pone.0333806

**Published:** 2026-03-24

**Authors:** Bingwen Wang, Wenhua Zha, Haifeng Lu

**Affiliations:** 1 East China University of Technology, School of Civil and Architectural Engineering, Nanchang, China; 2 Engineering Research Center for Geological Environment and Underground Space of Jiangxi Province, Nanchang, China; 3 Anhui University of Science and Technology, School of Earth and Environment, Huinan, China; Henan Polytechnic University, CHINA

## Abstract

The rational design and mechanical assessment of fault water-resisting coal pillars are essential for effective disaster prevention and mitigation. In the mechanical analysis of water-resisting coal (rock) pillars, deep-beam effects can significantly influence stress distribution, yet the applicability of existing analytical methods under deep-beam conditions has not been systematically compared or clearly defined. Classical elastic beam theory is widely used to evaluate pillar stresses, but it can yield substantial errors at non-slender geometries. Its limitations become pronounced when interlayer shear transfer and vertical compression cannot be neglected, which typically occurs when *h/L* > 0.2 (equivalently, *h/L* < 5).The aim of this paper is to compare the applicability and accuracy of analytical methods for deep-beam problems. We develop a layered deep-beam decomposition method, where the coal pillar–floor system is idealized as a simply supported rock beam under a uniformly distributed hydraulic load and discretized through the thickness into interacting shallow-beam layers to account for interlayer shear transfer and vertical compression. Based on a deep-beam model of the faulted floor, analytical solutions are obtained using both the classical elastic stress-function method and the proposed layered deep-beam decomposition method, and are validated against FLAC3D numerical simulations. Representative comparisons show that, for *h/L* = 0.3–1.0, the proposed approach (using 10, 16, and 20 layers) predicts mid-span normal stresses with relative errors of 6.0%–9.9%. In contrast, the classical elasticity solution deteriorates rapidly as h/L increases: the relative error can exceed 100% at larger *h*/*L*, and the solution fails to capture the downward migration of the neutral axis. Application to an engineering case from a deep coal mine in northern Anhui Province further indicates that, after incorporating a safety factor, the predicted pillar width is consistent with empirical design guidelines, supporting the method’s engineering applicability. Overall, this study focuses on the comparison of applicability and computational accuracy between the two analytical methods, which helps to clarify the applicable conditions and advantages of each method for deep-beam models in water-resisting coal pillar analysis.

## 1. Introduction

Water hazard prevention remains one of the most significant challenges in coal mine safety. Fault zones, karst conduits, and fractures associated with faults, as well as water-rich aquifers, can all pose serious threats to safe mining operations [1]. Despite sustained research efforts, accurately predicting and preventing fault-related water inrush remains challenging, and such incidents continue to occur [2]. A fault water-resisting (water-proof) coal pillar refers to the coal mass deliberately retained between the mined-out area and a water-conducting or water-bearing fault. Acting as a hydraulic barrier, it prevents hydraulic connection and reduces the risk of mine flooding [3]. Reliable mechanical assessment of these pillars is therefore essential for rational design and risk control.

In Poland, Hungary, and other countries affected by karst water in floor strata, extensive research has been conducted on the formation mechanisms and structural deformation of faulted rock masses [4–7]. Similarly, numerous inrush accidents in China have driven scholars to explore the design and retention of water-resisting coal pillars using mechanical models, stress analysis, and numerical simulations^.^ For example, Wang et al. [8] investigated the influence of mining-induced stress redistribution on fault zones and surrounding elastic strata and analyzed the relationship between coal pillar width and rock burst risks. Wu et al. [9] studied fault reactivation mechanisms and mining layouts with respect to fault orientation, proposing a conceptual model based on combined static and dynamic stress effects. Wang et al. [10] utilized in situ and laboratory data alongside FLAC3D numerical simulations to determine the appropriate width of water-resisting coal pillars in the F22 fault zone of the Qianyingzi Mine. Zhang et al. [11] systematically reviewed advances in pillar instability and failure analysis, synthesizing the principal theoretical, numerical, and in situ approaches for assessing the load–strength behavior of pillar support systems and outlining their respective applicability and limitations. Long et al. [12] developed a mechanical model for fault-induced inrush from aquifers and derived an analytical formula for calculating the distance from the coal seam floor to the fault intersection with the peak stress line. Yu et al. [13] proposed a fissure flow model based on the constant total energy equation, considering the spatial relationship between water-conducting faults and coal seams. Zhen et al. [14] enhanced the fault pillar model to more accurately evaluate internal static stress distribution and identify key influencing parameters.

In most of the above studies, coal pillars are idealized as shallow beams and analyzed using classical beam theory, such as the Euler–Bernoulli approach, under the assumption of plane sections [15–18]. This simplification is appropriate for slender members with a small thickness-to-span ratio (*h*/*L* < 0.2). When *h*/*L* ≥ 0.2, deep-beam behavior becomes significant and shear deformation can no longer be neglected [19]. In such cases, the Timoshenko beam theory [20] is more appropriate. Applying shallow-beam theory deep-beam conditions may result in substantial errors, particularly in high-stress deep fault zones. Although research has grown in areas such as fault structure analysis [21], inrush mechanisms [22], coal pillar width optimization [23], and early warning systems [24], studies focusing on the mechanical characteristics of deep beams remain limited. Moreover, classical analytical methods based on Euler–Bernoulli assumptions often neglect vertical compression and interlayer shear effects, which are crucial for accurately characterizing the mechanical response of deep beam-like coal pillars [19]. However, existing reviews indicate a clear gap in the systematic application of deep-beam theory to the mechanical analysis of coal pillars. Current studies on fault-induced water inrush and water-resisting coal pillars [11,25,26] mainly focus on fault structural characteristics, water-inrush mechanisms, pillar-width optimization, and early-warning technologies, with emphasis on elucidating the formation of seepage pathways and the evolution of water inrush under mining-induced disturbances. Meanwhile, the design and optimization of water-resisting coal pillars still rely primarily on numerical simulations, empirical criteria, or classical elastic analytical frameworks. Most of these studies concentrate on engineering calibration of pillar-width parameters and the development of instability criteria [27,28]. As a result, deep-beam effects are often discussed only qualitatively, and key deep-beam characteristics that may govern the response—such as shear deformation, vertical compression, and neutral-axis migration—cannot be reliably captured. This limitation, in turn, reduces the credibility and generalizability of stability assessment and width design for water-resisting coal pillars adjacent to faults. In coal pillar analysis, the transition from shallow-beam to deep-beam behavior introduces challenges that are rarely addressed in a systematic manner. Under hydraulic loading and strong rock-mass confinement, coal pillars exhibit pronounced through-thickness compression and interlayer shear transfer; therefore, shallow-beam (Euler–Bernoulli) assumptions may become invalid and key deep-beam responses are not captured, leaving a clear gap in design-oriented, verifiable stress solutions for fault-adjacent water-resisting pillars.

To address these gaps, this study introduces a deep-beam-based analytical framework for fault-adjacent water-resisting coal pillars and takes the comparison of method applicability as the primary research objective. Two analytical approaches are considered and contrasted. The first is the classical elasticity-based stress-function method, which provides a closed-form stress solution under conventional continuum assumptions and is employed here as a theoretical reference. The second is a layered deep-beam decomposition method proposed in this study, in which the deep beam is discretized through the thickness into interacting shallow-beam layers so that interlayer shear transfer and through-thickness compressive deformation can be explicitly represented. Based on the same deep-beam idealization, analytical solutions are derived using both methods and are then validated against FLAC3D numerical simulations. Finally, the framework is applied to an engineering case involving a faulted mine floor to demonstrate its design utility. Overall, this study clarifies the applicable conditions, advantages, and limitations of each analytical method for deep-beam modeling in water-resisting coal pillar analysis, providing practical guidance for method selection under non-slender geometries.

### 1.1. Development of the rock beam model for fault water-resisting coal pillars

**Fig 1:**
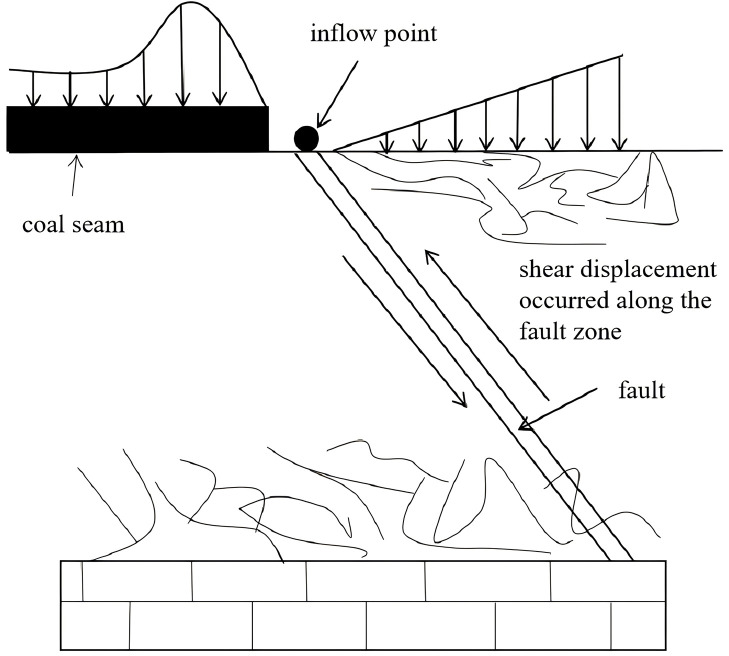
Schematic diagram of water inrush from fault.

During the mining process, fault-induced water inrush incidents frequently occur. On the one hand, the overburden pressure of the mine imposes stress on both sides of the fault, resulting in deformation or displacement of the surrounding rock. On the other hand, mining-induced disturbances lead to stress redistribution within the rock mass, which may cause the development of fault-related fractures and subsequently trigger floor water inrush, as illustrated in Fig 1.

**Fig 2:**
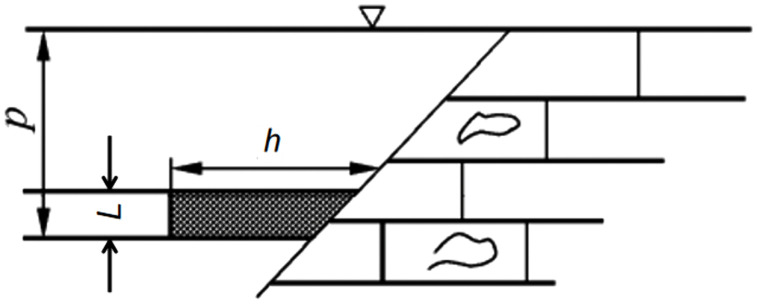
Layout of a fault water-resisting coal (rock) pillar: *p* represents the hydraulic pressure; *h* denotes the retained width of the coal pillar; *L* is the coal seam thickness or mining height.

To prevent fault-induced water inrush accidents, it is common practice to retain fault water-resisting coal pillars, making the determination of their appropriate width a critical issue, as illustrated in Fig 2. In order to address the coal pillar layout problem shown in Fig 2, the pillar is typically modeled as a beam subjected to water pressure, and its width is determined through mechanical stress analysis. This approach is also prescribed in the “Regulations for the Prevention and Control of Mine Water Hazards in Coal Mines” in China.However, the regulatory method treats the coal pillar as a simply supported beam under uniformly distributed water pressure, and applies shallow-beam theory to obtain the stress distribution and pillar width. In practice, the pillar width *h* is generally greater than the coal seam thickness *L*, which means the stress problem should be treated as a deep beam rather than a shallow one. Applying shallow-beam assumptions in such cases leads to discrepancies between analytical results and actual stress conditions.

When designing fault water-resisting coal pillars, factors such as fault dip angle, pillar width, coal seam thickness, and the aspect ratio between pillar width and seam thickness must be considered. These variables influence the stress redistribution in the surrounding strata during actual mining operations, and a comprehensive assessment is essential for the rational retention of fault barrier pillars. Due to the inherent limitations of theoretical solutions, the deep-beam model adopted in this study introduces simplifications by neglecting the influence of the fault dip angle. Given the prevalence of fissures in coal-bearing rock masses, treating the ends of the rock beam as pinned supports is a practical and effective assumption [29,30]. Accordingly, the water-resisting coal pillar beneath the fault zone subjected to confined water pressure is modeled as a simply supported deep beam. Deep-beam theory is then applied to investigate the stress distribution and failure behavior of the coal seam floor, thereby providing a theoretical foundation for water inrush prediction.

**Fig 3:**
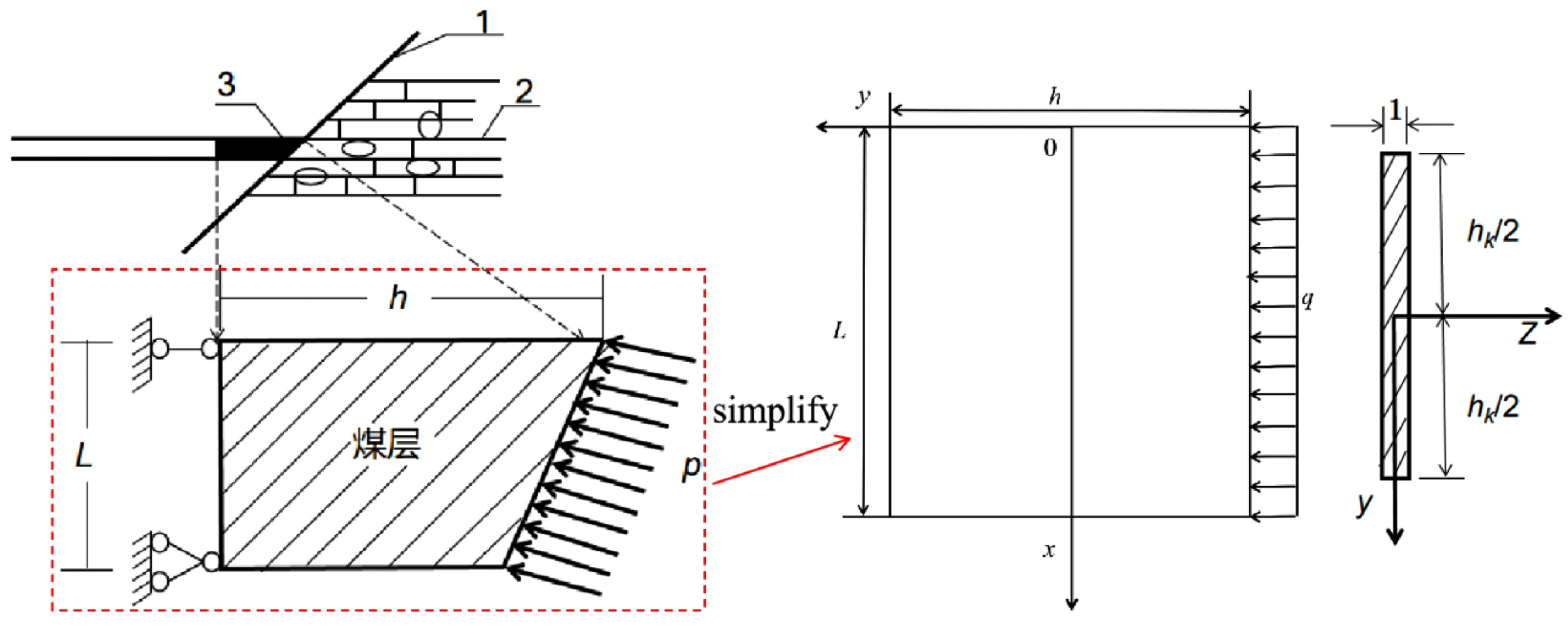
Mechanical model transformation of the fault water-resisting coal pillar:1: Water-conducting fault; 2: Water-bearing aquifer; 3: Coal seam; *h*: Retained width of the coal pillar; *L*: Coal seam thickness or mining height; *p*: Hydraulic pressure.

In practical engineering, the design of fault water-resisting coal pillars should be based on geological conditions, hydrostatic pressure, and fault geometry. Various types of barrier pillars exist, but this chapter focuses on the case where the coal seam is in direct contact with a water-bearing fault. The mechanical model considers the influence of water pressure on the coal floor and is established as a simply supported deep beam, as shown in Fig 3. This simplified model is particularly suitable for steeply dipping faults. Accordingly, the main objective of this paper is to compare the applicability and accuracy of these two analytical methods for fault water-resisting coal pillars under deep-beam conditions. The coordinate system is defined with the origin *O* at the midpoint of the upper surface of the key layer. The vertical axis *y* corresponds to the beam height, and the horizontal axis *x* corresponds to the beam length. The right-hand side of the beam is subjected to a uniformly distributed water pressure *p*. In the model, *L* denotes the coal seam thickness, *h* is the retained width of the pillar, the floor is assumed to have a unit width of 1, and the average unit weight of the coal seam floor is γ.

For convenience in subsequent analysis, the aspect ratio is defined as the ratio of the pillar width to the coal seam thickness (*h/L*). Building upon this foundation, this paper employs two analytical methods to solve the aforementioned deep-beam problem: the elastic mechanics stress function method is used to obtain the benchmark analytical stress solution for comparison; the layered deep-beam decomposition method discretizes the deep beam along its thickness into several mutually coupled shallow-beam layers to reflect interlayer shear transfer and vertical compression effects, while allowing precision control through the number of layers. Subsequently, the results from both methods are compared to evaluate solution accuracy. Furthermore, the influence of aspect ratio and the number of layers on the computational accuracy of the layered deep-beam decomposition method is analyzed.

## 2. Analytical solutions and accuracy evaluation of two solution methods

### 2.1. Stress function method

The stress function method is one of the commonly used approaches in elasticity theory for solving beam-related problems. This method derives the stress function expressions based on the compatibility conditions, under the premise that the differential equations of equilibrium and strain compatibility are satisfied. According to the formulation presented in [31], the stress function relationshipis given by:


$$\begin{array}{c} u=\frac{\text{1}}{E}[-\frac{2q}{h^{\text{3}}}x^{\text{3}}y+x\left(\frac{4qy^{\text{3}}}{h^{\text{3}}}-\frac{qy}{15h}\right)-\mu x\left(-\frac{q}{\text{2}}+\frac{3qy}{2h}-\frac{2qy^{\text{3}}}{h^{\text{3}}}\right)-\omega y+u_{o}\backslash \nu =\frac{\text{1}}{E}\left[-\mu \left(-\frac{3q}{h^{\text{3}}}x^{\text{2}}y^{\text{2}}+\frac{q}{h^{\text{3}}}y^{\text{4}}-\frac{q}{30h}y^{\text{2}}\right)-\frac{q}{\text{2}}y+\frac{3q}{4h}y^{\text{2}}-\frac{qy^{\text{4}}}{2h^{\text{3}}}+\frac{2q}{h^{\text{3}}}x^{\text{3}}-\frac{\left(45\mu -88\right)}{30h}qx+\omega x+\nu_{\text{0}}\right] \end{array}\}$$ (1)

In Eq. (1),$u_{\text{0}}$ and $\nu_{\text{0}}$rrepresent the horizontal and vertical displacements at a given point on the model, respectively, both of which are equal to zero;ω denotes the rotational displacement at that point.All of them are integration constants.in which, $\omega =\frac{\left(45\mu -88\right)q}{30h}-\frac{2ql^{\text{2}}}{h^{\text{3}}}$

### 2.2. Layered deep-beam decomposition method

In layered deep-beam decomposition method, several analytical approaches have been developed, including the state-space method [32], the bar mechanics method [33], and series solution techniques [34]. However, systematic studies on the accuracy of stress distribution predictions and the evolution of the neutral axis with changing structural parameters remain limited. Traditional beam theory typically neglects the interlayer compressive stress *σ*_*y*_ in shallow-beam models, an assumption that becomes increasingly invalid in deep beams with large aspect ratios, thereby restricting its applicability under complex stress conditions.

To accurately characterize the non-uniform stress response of deep fault water-resisting coal pillars under hydraulic loading, this study extends the previously developed layered beam decomposition theory [31], adapting it to the structural configuration and mechanical behavior of fault-controlled pillars. In this method, the deep beam is discretized through the thickness into *N* interacting layers. Each layer is modeled as a shallow beam spanning the full length, and interlayer coupling is introduced to represent shear transfer and through-thickness compression.The approach captures key mechanical features of deep coal pillars subjected to overburden loading and fault-induced shear disturbances, including neutral axis migration, interlayer bending mismatch, and shear stress nonuniformity. The pillar is equivalently divided into a series of layers with equal thickness, and integral expressions are derived for shear stress and axial displacement in each layer. This enables a detailed representation of shear force transfer and multi-layer synergistic deformation along the pillar height under fault influence. Compared to traditional homogeneous elasticity theory, the proposed method offers significant advantages in revealing the mechanisms behind stress concentration and amplified deformation in fault-adjacent coal pillars. It provides a robust analytical framework for evaluating the bearing capacity and water inrush risk of water-resisting coal pillars in deep, structurally complex environments.

**Fig 4:**
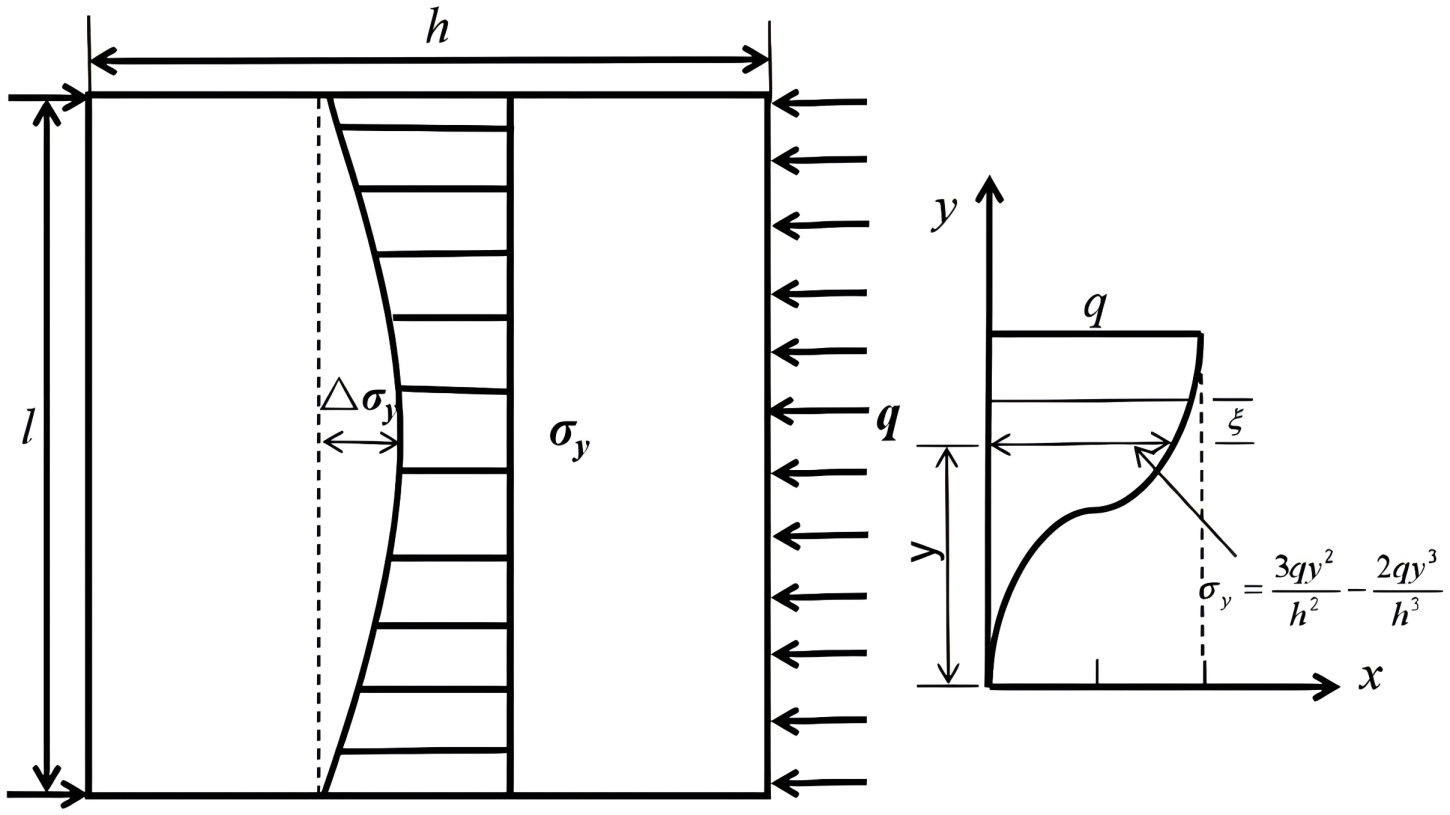
Shows the distribution of *σ*_*y.*_


According to the layered beam decomposition approach, the loading on each shallow-beam segment corresponds to the interlayer compressive stress *σ*_*y*_. Due to the uniformly distributed load *q* acting on the topmost shallow beam, the bending moment in each layer increases progressively from top to bottom. As shown in Fig 4, based on elastic solutions for shallow beams, the stress components induced by the distributed load *q* can be derived as follows:

$$\sigma_{y}=\frac{3qy^{\text{2}}}{h^{\text{2}}}-\frac{2qy^{\text{3}}}{h^{\text{3}}}$$ (2)

Due to the influence of the interlayer compressive stress *σ*_*y*_, the tensile and compressive deformations on the upper and lower surfaces of each segmented “shallow beam” differ. In this case, the average value of the deformation is used for calculation. After the deep beam is divided, the displacements at the mid-span point on both the upper and lower surfaces of each shallow beam segment are assumed to be equal.Let *w*_*u*_ and *w*_*l*_ denote the displacements of the upper and lower surfaces, respectively, where the superscript mmm indicates the displacement at the beam’s mid-span and *s* refers to the beam end. The displacement caused by bending deformation is represented by *w*_*b*_. Based on this, the displacement relationship between the upper and lower surfaces of the segmented shallow beam can be expressed as follows:


$$w_{ib}=w_{\left(i+1\right)b}+\frac{3q}{2E}\left\{\int_{i}\left[1-\left(\frac{3y^{\text{2}}}{h^{\text{2}}}-\frac{2y^{\text{3}}}{h^{\text{3}}}\right)\right]dy+\int_{i+1}\left[1-\left(\frac{3y^{\text{2}}}{h^{\text{2}}}-\frac{2y^{\text{3}}}{h^{\text{3}}}\right)\right]dy\right\}$$ (3)

In the equation, w_*ib*_、w_*il*_、w_*iu*_ (*i* = 1, 2, 3....)denote the bending-induced displacement, the upper surface displacement, and the lower surface displacement, respectively, for the iii-th shallow-beam layer (counted from top to bottom) in an *i*-layer decomposition of the deep beam, with units in meters (m).； $w_{i}^{m}$、$w_{i}^{s}$ (*i* = 1、2、3....) denote the displacements at the mid-span and at the ends of the iii-th shallow-beam layer, respectively, counted from top to bottom in an *i*-layer decomposition of the deep beam.Using the above relationships, the displacements of all other layers can be determined accordingly. According to the formulation presented in [26], the bending-induced displacement can be calculated as follows:


$$w_{ib}=\alpha_{i}\frac{\sigma_{i-1}+\Delta \sigma_{i-1}-\sigma_{i}-\Delta \sigma_{i}}{\xi E}l^{\text{2}}$$ (4)

In the equation，$\alpha_{i}$ (*i* = 1, 2, 3....)is the displacement coefficient induced by bending in the *i*-th shallow-beam layer obtained from the layered decomposition of the deep beam.；$\overset{―}{\alpha_{i,i+\text{1}}}$ denotes the average bending-induced displacement coefficient between two adjacent shallow beam layers, defined as: $\frac{\overset{―}{\alpha_{i,i+\text{1}}}=\left(\alpha_{i}+\alpha_{i+\text{1}}\right)}{\text{2}}$

The interlayer stress differences *△σ*_*i*_ for each water-resisting key layer are calculated accordingly. A system of equations is then established based on the moment equilibrium induced by the applied load. By solving this system using MATLAB, the stress distribution in each layer can be obtained. The same approach applies to the shear stress component in the internal force system of the deep beam. During the calculation, each layer is further treated as a shallow beam along the span, and the span may be divided into segments for integration and stress evaluation. This spanwise segmentation is introduced for computational convenience and does not change the primary through-thickness layering of the method. The distribution of shear stress is then derived by considering both the uniformly distributed load *q* and the disturbance effect of interlayer compressive stress *σ*_*y*_ on each segmented beam. The details of this process are omitted here for brevity.

To evaluate the accuracy of the layered deep-beam decomposition method and examine the influence of the number of layers on the analytical solution, a comparative analysis is conducted based on the mechanical model shown in Fig 3. Given that the layered solution accuracy depends on both the aspect ratio and discretization, an explicit error assessment is necessary before the method is used for pillar-width design. The proposed method is compared with both the conventional analytical solution from elasticity theory and the numerical results obtained using FLAC3D. The relative error is defined as the deviation between the analytical results and the numerical solution, and is used to assess the relationship between the calculated stress, the number of layers, and the aspect ratio. This analysis aims to reveal the accuracy characteristics of the decomposition method under varying conditions.

Let λ_1_, λ_2_, and λ_3_ denote the analytical solutions obtained from the layered deep-beam decomposition method with 10, 16, and 20 layers, respectively, and λ_4_ denote the solution based on conventional elasticity theory. The span of the beam is set to *L* = 10 m, and the beam heights *h* are varied as 3, 4, 5, 6, 7, 8, 9, and 10 m, corresponding to aspect ratios *h/L* = 0.3 to 1.0. The beam is subjected to a uniformly distributed load of *q* = 0.5 MPa, and the Poisson’s ratio is taken as 0.25.

### 2.3. Accuracy evaluation

**Fig 5:**
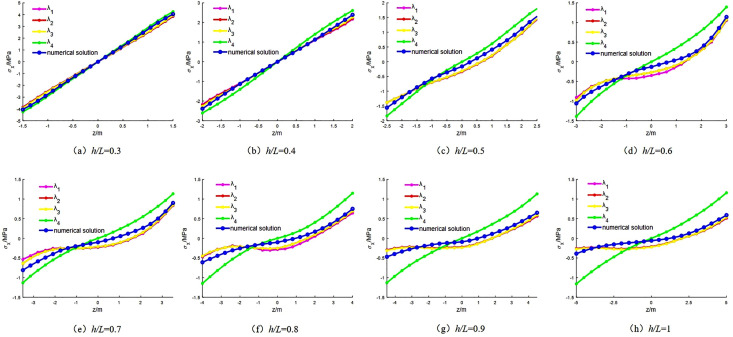
Comparison of mid-span stress when *h*/*L* = 0.5 ~ 1.

As shown in Fig 5, under varying aspect ratios (*h*/*L* = 0.5 to 1.0), the axial stress distribution along the *z*-direction at the beam mid-span exhibits a consistent trend, indicating that both analytical methods capture the overall stress variation pattern. However, with increasing aspect ratio, the stress deviation between the elasticity-based analytical solution and the numerical solution gradually increases, despite the neutral axis in the elastic solution remaining at the mid-height of the section. This deviation is primarily attributed to the fact that the classical elastic method neglects the interlayer compressive stress *σ*_*y*_, which plays a significant role in deep-beam mechanics.

During the loading process of a deep beam, substantial internal stress redistribution occurs. The interlayer compressive stress tends to concentrate downward toward the support region, leading to localized shear stress concentration and a downward shift of the neutral axis. In contrast, the layered deep-beam decomposition method more accurately reflects the actual stress state. Its results align well with the trends observed in numerical simulations and effectively capture the progressive downward migration of the neutral axis with increasing aspect ratio.

Moreover, as the number of layers increases, the layered deep-beam decomposition method exhibits a noticeable increase in accuracy. Nevertheless, under high aspect ratio conditions, a degree of error growth is still observed, suggesting that the accuracy of the decomposition is significantly influenced by the layer discretization strategy.

As shown in Table 1, the relative error increases from 4.3% to over 100%. This substantial deviation stems from the simplification *σ*_*y*_ = 0 adopted in the solution of the stress function and compatibility equations, which is no longer valid under deep mining conditions, where surrounding rock stress is highly complex and shear effects are intensified. Therefore, the elasticity-based solution is more suitable for shallow-buried beam structures. On the other hand, the accuracy of the layered deep-beam decomposition method improves with finer layer discretization, though the rate of improvement diminishes. This indicates that, in practical engineering applications, the number of layers should be chosen appropriately to avoid excessive error accumulation due to overly coarse discretization.

**Table 1:** Comparative analysis of normal stress between analytical and numerical solutions.

*h*/*L*	Numerical solution/ MPa	Layered beam decomposition/ MPa		Relative error/%	Elasticity theory/ MPa	Relative error/%
0.3	4.05	10	3.891	7.8	4.22	4.3
		16	3.923	6.1		
		20	3.942	6.0		
0.4	2.38	10	2.240	8.5	2.61	9.66
		16	2.271	6.8		
		20	2.280	6.6		
0.5	1.58	10	1.470	6.69	1.79	13.3
		16	1.491	5.63		
		20	1.490	5.69		
0.6	1.14	10	1.056	7.32	1.35	18.4
		16	1.069	6.23		
		20	1.070	6.14		
0.7	0.91	10	0.834	7.98	1.23	35.2
		16	0.846	7.13		
		20	0.849	6.70		
0.8	0.75	10	0.687	8.32	1.15	53.3
		16	0.691	7.87		
		20	0.694	7.50		
0.9	0.65	10	0.592	8.91	1.11	92.3
		16	0.596	8.31		
		20	0.597	8.15		
1	0.59	10	0.531	9.98	1.16	>100
		16	0.536	9.15		
		20	0.537	8.98		

Overall, the above comparisons confirm that the layered deep-beam method maintains stable accuracy over a wider range of aspect ratios than the classical elasticity solution, while allowing a practical accuracy–efficiency trade-off via the layer number. With this validation, Section 3 applies the proposed framework to a representative fault-controlled mine block to demonstrate its engineering workflow and design implications.

## 3. Engineering application case study

The studied mine, affiliated with the Wanbei Coal and Power Group, is located in the central region of the Huaibei Plain, where the terrain is relatively flat. The surface elevation ranges from +19.68 m to +24.72 m, generally around +23 m, showing a gradual decline from the northwest to the southeast. The average burial depth of the coal mining face is approximately 850 m. The southern boundary of the mine is defined by exploration lines and fault zones, while the northern extent reaches the surface projection of the −1200 m contour line of the mining coal seam.A total of 10 faults with throw ≤ 20 m have been identified. These faults are primarily aligned in the NE direction, consistent with the orientation of the major fault, and are concentrated near it. Their development exerts a significant influence on coal seam extraction. Localized folds have also formed in some areas due to fault drag effects in the vicinity of the major fault.

In one of the working faces, the main roadway is 2,196 m long with an elevation ranging from −850 m to −810 m, while the air-return roadway extends 2,252 m, with an elevation between −830 m and −770 m. Both roadways are oriented at an azimuth of 339°. The inclined opening cut is 200 m in length, with a horizontal projection distance of 194 m. The average dip angle of the coal seam in the working face is 18°. In this area, the coal seam is relatively stable, with an average thickness M = 2 m and minor variations. The minability index of the seam is K = 98.7. The characteristics of the roof and floor strata in the working face are summarized in Table 2.

**Table 2:** Table of roof and floor of coal seam in working face.

Stratigraphic Position	Name	Lithology	Thickness (m)	Lithological characteristics
Roof	Main roof	Fine sandstone	2.29	Fine-grained texture, primarily composed of quartz and feldspar, locally argillaceous, with siliceous cementation.
	Immediate roof	Mudstone, carbonaceous mudstone, siltstone	4.57	
	False roof	Mudstone		
Floor	Immediate floor	Mudstone	0.70	Argillaceous structure, massive, uneven fracture surfaces
	Main floor	Siltstone	1.58	Silt-rich texture, massive, dense, hard, contains fossil fragments

**Fig 6:**
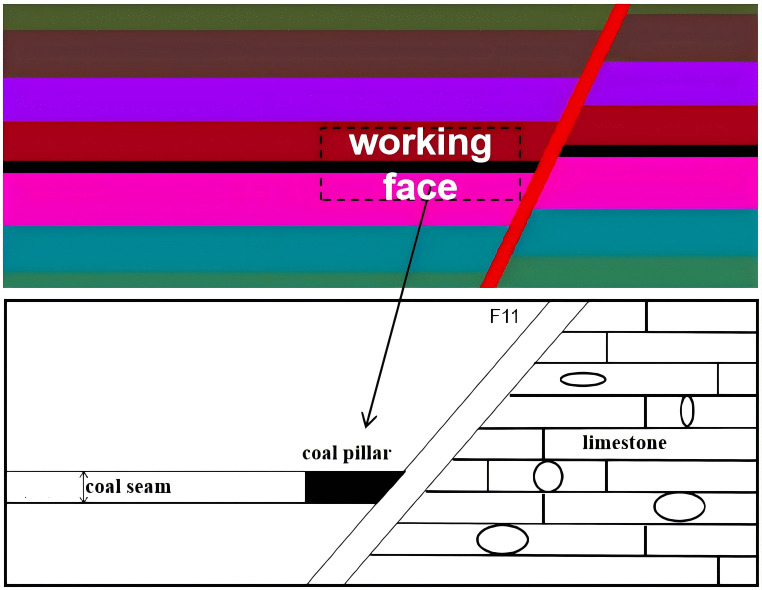
Schematic diagram of working face fault.

**Fig 7:**
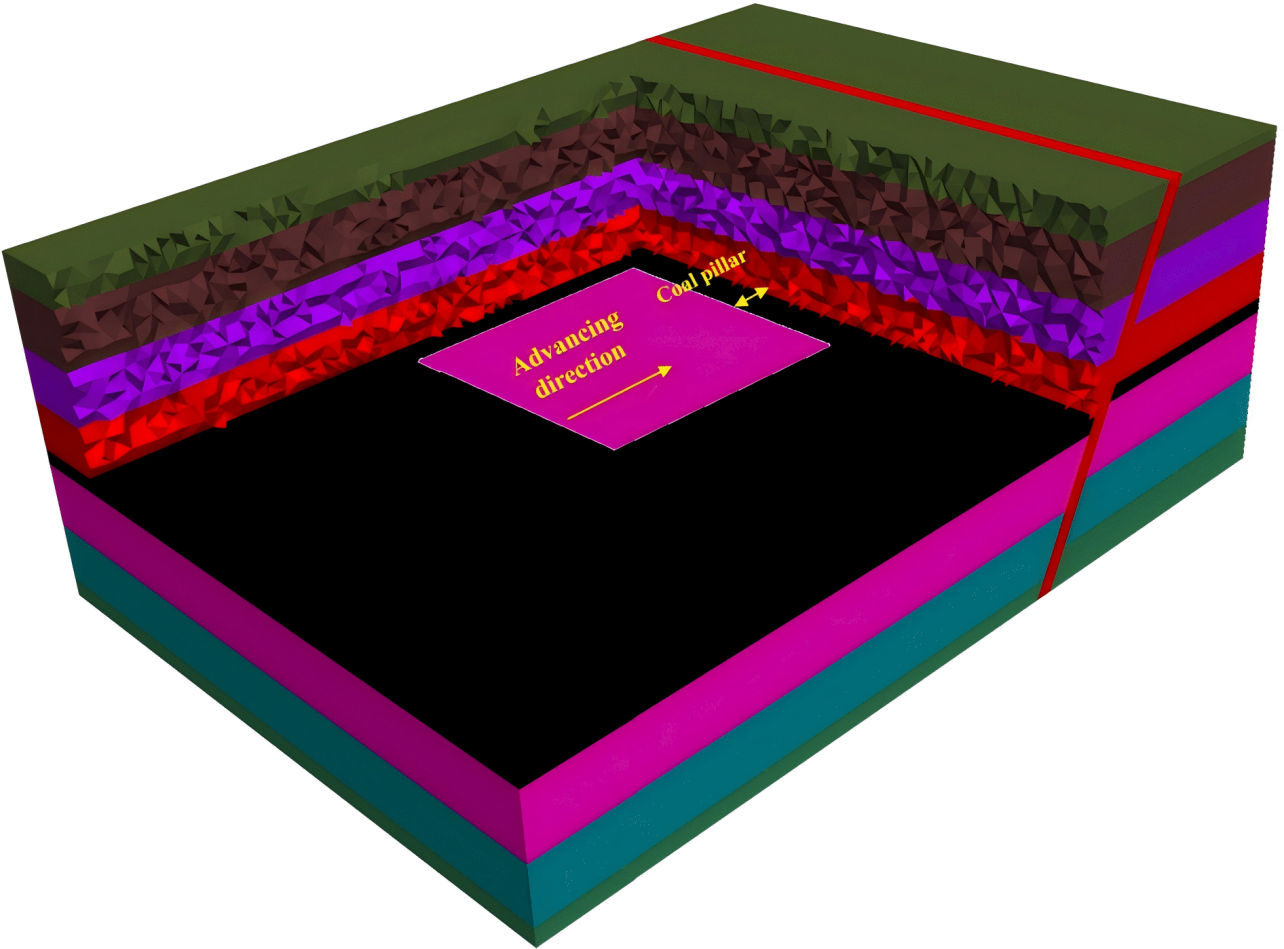
Numerical simulation of the water-resisting key stratum in the working face.

A normal fault, designated F11, is present near the working face. This fault has a significant throw, resulting in the juxtaposition of coal-bearing strata in the hanging wall against the water-bearing limestone aquifer of the Taiyuan Formation in the footwall, as shown in Figs 6 and 7.

Exploration and in situ measurements indicate that most faults within the mine exhibit weak water richness and poor water conductivity. However, during roadway excavation through fault zones, groundwater within fault-related fracture zones can enter the mine, disrupting the original hydrogeological balance and enhancing the fault’s water-conducting capacity. If the main mining coal seam becomes directly connected with a water-rich aquifer, the risk of water inrush significantly increases.

Fault F11 causes direct contact between the coal seam and the Taiyuan Formation limestone. Given the strong water-rich characteristics and high hydraulic pressure of the Taiyuan limestone, this poses a serious threat to coal seam extraction in the working face. In this chapter, a deep-beam model is established to analyze the layout of the water-resisting coal pillar for safe retreat mining in this fault-affected block. The coal pillar is subjected to a static water pressure of *p* = 1 MPa, and the average tensile strength of the coal seam is 0.8 MPa.

Accurately calculating the required width of the water-resisting coal pillar near this fault is of great importance for ensuring mining safety and preventing fault-induced water inrush accidents.

In this study, the design of the water-resisting coal pillar associated with fault F11 in the working face focuses primarily on the pillar subjected to compressive loading along the bedding direction. The pillar width is commonly determined using the following calculation formula:


$$\text{h}=0.5KM\sqrt{\frac{3P}{K_{p}}}$$ (5)

Where: *h* is thewidth of the fault water-resisting coal pillar (m); *L* is thecoal seam thickness or mining height (m), taken as 2 m; *K*_p_ is thetensile strength of coal (MPa), taken as 0.8 MPa; *p* is the hydraulic pressure (MPa), taken as 1 MPa; *K* is the safety factor, generally ranging from 2 to 5; a value of 3 is adopted in this design.

This formula is also specified in the “Regulations for the Prevention and Control of Water Hazards in Coal Mines” and is derived based on a simply supported beam model using conventional material mechanics theory [35].By substituting the above parameters into the formula, the required width of the fault water-resisting coal pillar is calculated as:


$$\text{h}=0.5\times 3\times 2\times \sqrt{\frac{3\times 100}{80}}=5.81m$$

**Fig 8:**
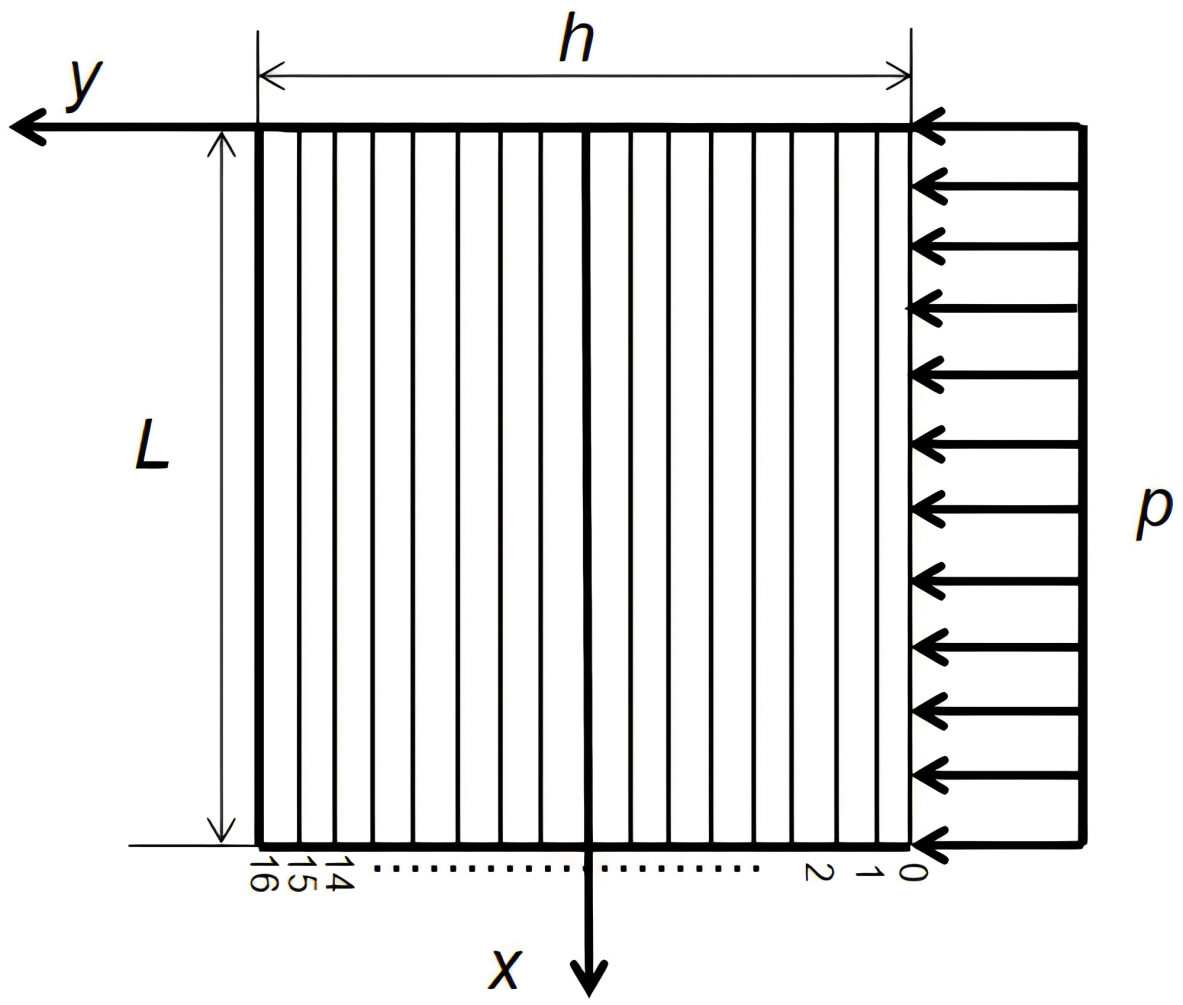
Layered model of deep beam of fault water-resisting coal pillar.

Since the coal seam thickness is generally much smaller than the retained width of the coal pillar, the structure can be equivalently modeled as a deep beam. Therefore, deep-beam theory must be applied to accurately calculate the maximum tensile stress and better reflect actual stress conditions.The following analysis examines the deep-beam behavior of the fault water-resisting coal pillar under different retained widths: *h* = 1.6 m, 1.8 m, 2.0 m, 4.0 m, and 6.0 m. The results are evaluated using the deep beam theory and compared with corresponding numerical solutions. A schematic diagram of the layered deep-beam decomposition method for the fault coal pillar is shown in Fig 8.

Taking the case where the coal pillar width h = 1.6 m as an example, the model is discretized into 16 layers for the layered beam decomposition. The same approach is applied to other pillar widths and is therefore not repeated here.

For the first layer, the calculation is as follows:


$$\int_{\text{1}}\frac{3{\text{y}}^{\text{2}}}{{\text{h}}^{\text{2}}}-\frac{2{\text{y}}^{\text{3}}}{{\text{h}}^{\text{3}}}dy=\frac{\text{1}}{{\text{h}}^{\text{2}}}y^{\text{3}}{|}_{\text{0}}^{1.6/16}-\frac{\text{1}}{{\text{2h}}^{\text{3}}}y^{\text{4}}{|}_{\text{0}}^{1.6/16}=0.00037$$ (6)

Similarly, it can be obtained that:


$$\begin{array}{c} \int_{\text{2}}\frac{3{\text{y}}^{\text{2}}}{{\text{h}}^{\text{2}}}-\frac{2{\text{y}}^{\text{3}}}{{\text{h}}^{\text{3}}}=0.00255,\int_{\text{3}}\frac{3{\text{y}}^{\text{2}}}{{\text{h}}^{\text{2}}}-\frac{2{\text{y}}^{\text{3}}}{{\text{h}}^{\text{3}}}=0.00663,\int_{\text{4}}\frac{3{\text{y}}^{\text{2}}}{{\text{h}}^{\text{2}}}-\frac{2{\text{y}}^{\text{3}}}{{\text{h}}^{\text{3}}}=0.01232\\ \int_{\text{5}}\frac{3{\text{y}}^{\text{2}}}{{\text{h}}^{\text{2}}}-\frac{2{\text{y}}^{\text{3}}}{{\text{h}}^{\text{3}}}=0.01933,\int_{\text{6}}\frac{3{\text{y}}^{\text{2}}}{{\text{h}}^{\text{2}}}-\frac{2{\text{y}}^{\text{3}}}{{\text{h}}^{\text{3}}}=0.02736,\int_{\text{7}}\frac{3{\text{y}}^{\text{2}}}{{\text{h}}^{\text{2}}}-\frac{2{\text{y}}^{\text{3}}}{{\text{h}}^{\text{3}}}=0.03612\\ \int_{\text{8}}\frac{3{\text{y}}^{\text{2}}}{{\text{h}}^{\text{2}}}-\frac{2{\text{y}}^{\text{3}}}{{\text{h}}^{\text{3}}}=0.04533,\int_{\text{9}}\frac{3{\text{y}}^{\text{2}}}{{\text{h}}^{\text{2}}}-\frac{2{\text{y}}^{\text{3}}}{{\text{h}}^{\text{3}}}=0.05468,\int_{10}\frac{3{\text{y}}^{\text{2}}}{{\text{h}}^{\text{2}}}-\frac{2{\text{y}}^{\text{3}}}{{\text{h}}^{\text{3}}}=0.06388\\ \int_{11}\frac{3{\text{y}}^{\text{2}}}{{\text{h}}^{\text{2}}}-\frac{2{\text{y}}^{\text{3}}}{{\text{h}}^{\text{3}}}=0.07265,\int_{12}\frac{3{\text{y}}^{\text{2}}}{{\text{h}}^{\text{2}}}-\frac{2{\text{y}}^{\text{3}}}{{\text{h}}^{\text{3}}}=0.08068,\int_{13}\frac{3{\text{y}}^{\text{2}}}{{\text{h}}^{\text{2}}}-\frac{2{\text{y}}^{\text{3}}}{{\text{h}}^{\text{3}}}=0.08768\\ \int_{14}\frac{3{\text{y}}^{\text{2}}}{{\text{h}}^{\text{2}}}-\frac{2{\text{y}}^{\text{3}}}{{\text{h}}^{\text{3}}}=0.09337,\int_{15}\frac{3{\text{y}}^{\text{2}}}{{\text{h}}^{\text{2}}}-\frac{2{\text{y}}^{\text{3}}}{{\text{h}}^{\text{3}}}=0.09745,\int_{16}\frac{3{\text{y}}^{\text{2}}}{{\text{h}}^{\text{2}}}-\frac{2{\text{y}}^{\text{3}}}{{\text{h}}^{\text{3}}}=0.09962 \end{array}\}$$ (7)

The displacement relationship for each layer is given by:


$$\begin{array}{c} {\text{w}}_{1\text{b}}=w_{2b}+2.956\frac{\xi }{E},w_{2\text{b}}=w_{3b}+2.862\frac{\xi }{E},w_{3\text{b}}=w_{4b}+2.716\frac{\xi }{E}\\ w_{4\text{b}}=w_{5b}+2.525\frac{\xi }{E},w_{5\text{b}}=w_{6b}+2.300\frac{\xi }{E},w_{6\text{b}}=w_{7b}+2.048\frac{\xi }{E}\\ w_{7\text{b}}=w_{8b}+1.778\frac{\xi }{E},w_{8\text{b}}=w_{9b}+1.500\frac{\xi }{E},w_{9\text{b}}=w_{10b}+1.222\frac{\xi }{E}\\ w_{1\text{0b}}=w_{11b}+0.952\frac{\xi }{E},w_{1\text{1b}}=w_{12b}+0.700\frac{\xi }{E},w_{1\text{2b}}=w_{13b}+0.475\frac{\xi }{E}\\ w_{1\text{3b}}=w_{14b}+0.284\frac{\xi }{E},w_{1\text{4b}}=w_{15b}+0.138\frac{\xi }{E},w_{1\text{5b}}=w_{16b}+0.044\frac{\xi }{E} \end{array}\}$$ (8)

When the model is divided into 16 layers, the interlayer stress differences *△σ*_*i*_ for the water-resisting key strata are calculated based on the formulation provided in reference [31]. Meanwhile, a moment equilibrium equation is established under the applied load, leading to the following relationship:


$$\begin{array}{c} 0.2672\Delta \sigma_{\text{0}}+\Delta \sigma_{\text{1}}+2\Delta \sigma_{\text{2}}+3\Delta \sigma_{\text{3}}+4\Delta \sigma_{\text{4}}+5\Delta \sigma_{\text{5}}+6\Delta \sigma_{\text{6}}+\Delta \sigma_{\text{7}}\\ +8\Delta \sigma_{\text{8}}+9\Delta \sigma_{\text{9}}+10\Delta \sigma_{10}+11\Delta \sigma_{11}+12\Delta \sigma_{12}+13\Delta \sigma_{13}+14\Delta \sigma_{14}\\ +15\Delta \sigma_{15}+7.7328\Delta \sigma_{10}=0 \end{array}$$ (9)

$$\begin{array}{c} \Delta \sigma_{\text{0}}+2(\Delta \sigma_{\text{1}}+\Delta \sigma_{\text{2}}+\Delta \sigma_{\text{3}}+\Delta \sigma_{\text{4}}+\Delta \sigma_{\text{5}}+\Delta \sigma_{\text{6}}+\Delta \sigma_{\text{7}}+\Delta \sigma_{\text{8}}+\Delta \sigma_{\text{9}}\\ +\Delta \sigma_{10}+\Delta \sigma_{11}+\Delta \sigma_{12}+\Delta \sigma_{13}+\Delta \sigma_{14}+\Delta \sigma_{15})+\Delta \sigma_{16}=0 \end{array}$$ (10)

By establishing a system of equations through simultaneous formulation, the resulting equations are solved using MATLAB, yielding the following results:


$$\begin{array}{c} \sigma_{\text{0}}=1.48,\text{\textbackslash{}hspace\{0.17em}\;\}\sigma_{\text{1}}=0.95,\text{\textbackslash{}hspace\{0.17em}\;\}\sigma_{\text{2}}=0.51,\text{\textbackslash{}hspace\{0.17em}\;\}\sigma_{\text{3}}=0.25\\ \sigma_{\text{4}}=0.16,\text{\textbackslash{}hspace\{0.17em}\;\}\sigma_{\text{5}}=0.02,\text{\textbackslash{}hspace\{0.17em}\;\}\sigma_{\text{6}}=0.008,\text{\textbackslash{}hspace\{0.17em}\;\}\sigma_{\text{7}}=-0.024\\ \sigma_{\text{8}}=-0.093,\text{\textbackslash{}hspace\{0.17em}\;\}\sigma_{\text{9}}=-0.133,\text{\textbackslash{}hspace\{0.17em}\;\}\sigma_{10}=-0.181,\text{\textbackslash{}hspace\{0.17em}\;\}\sigma_{11}=-0.264\\ \sigma_{12}=-0.198,\text{\textbackslash{}hspace\{0.17em}\;\}\sigma_{13}=-0.105,\text{\textbackslash{}hspace\{0.17em}\;\}\sigma_{14}=-0.203,\text{\textbackslash{}hspace\{0.17em}\;\}\sigma_{15}=-0.348\\ \sigma_{16}=-0.541 \end{array}\}MPa$$

**Fig 9:**
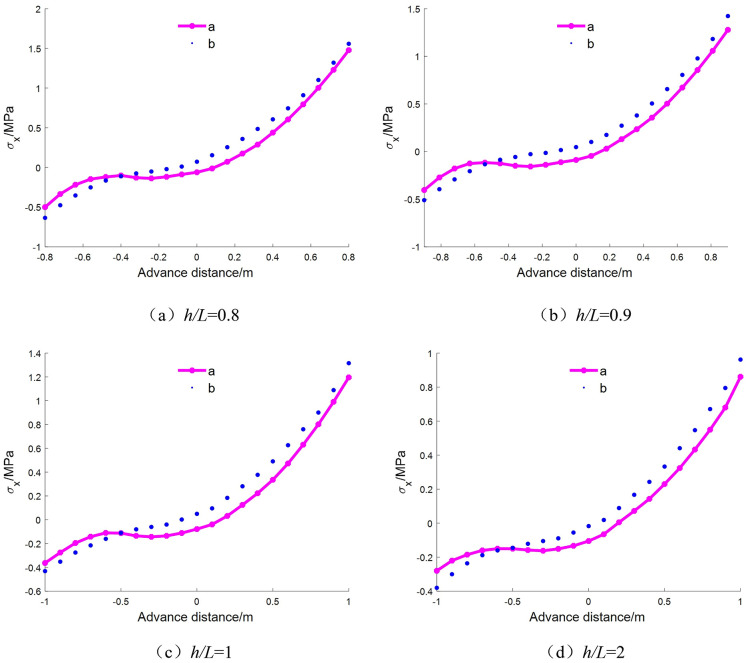
The stress change of coal floor when *h/L* = 0.8 ~ 2.

Based on the deep-beam calculation method, the stress results for all aspect ratios can be obtained. To verify the reliability of the analytical solution derived from the layered deep-beam decomposition theory, stress results from both the analytical method and FLAC3D numerical simulations are plotted using MATLAB for comparison.In this comparison, the analytical solution from the layered deep-beam decomposition method is referred to as solution a, and the numerical solution from FLAC3D is referred to as solution b. The critical width of the fault water-resisting coal pillar is then estimated based on the tensile strength threshold of the coal seam.According to the geological data, the coal pillar is subjected to a uniformly distributed vertical load of 1 MPa applied along the fault plane. The empirical formula provides an initial estimate of the pillar width. For simplicity, a static parametric study is performed by varying the retained coal pillar width ℎ from 1.6 m upward in increments of 0.2 m. Here, ‘increment’ refers to the assumed pillar-width values used in independent calculations, rather than a time-dependent mining advance. At each step, stress values are calculated and compared against the FLAC3D numerical results.Assuming a coal seam thickness *L* = 2 m, the corresponding aspect ratio (*h*/*L*) starts at 0.8 and increases progressively. The calculation continues until the theoretical tensile stress is less than or equal to the coal’s tensile strength threshold of 0.8 MPa, as illustrated in Fig 9.

As shown in Fig 9, when the aspect ratio is 0.8 (Fig 9a), corresponding to a coal pillar width of 1.6 m with a layer thickness of 0.1 m, the maximum tensile stress in the coal pillar obtained from the layered deep-beam method solution is 1.478 MPa. For an aspect ratio of 0.9 (Fig 9b), i.e., a coal pillar width of 1.8 m and layer thickness of 0.1125 m, the maximum tensile stress is 1.278 MPa.When the aspect ratio reaches 1.0 (Fig 9c), corresponding to a pillar width of 2.0 m and layer thickness of 0.125 m, the maximum tensile stress is 1.196 MPa. At an aspect ratio of 2.0 (Fig 9d), i.e., a pillar width of 4.0 m and layer thickness of 0.25 m, the maximum tensile stress inside the coal pillar is 0.821 MPa, which exceeds the average tensile strength of the coal seam.

**Fig 10:**
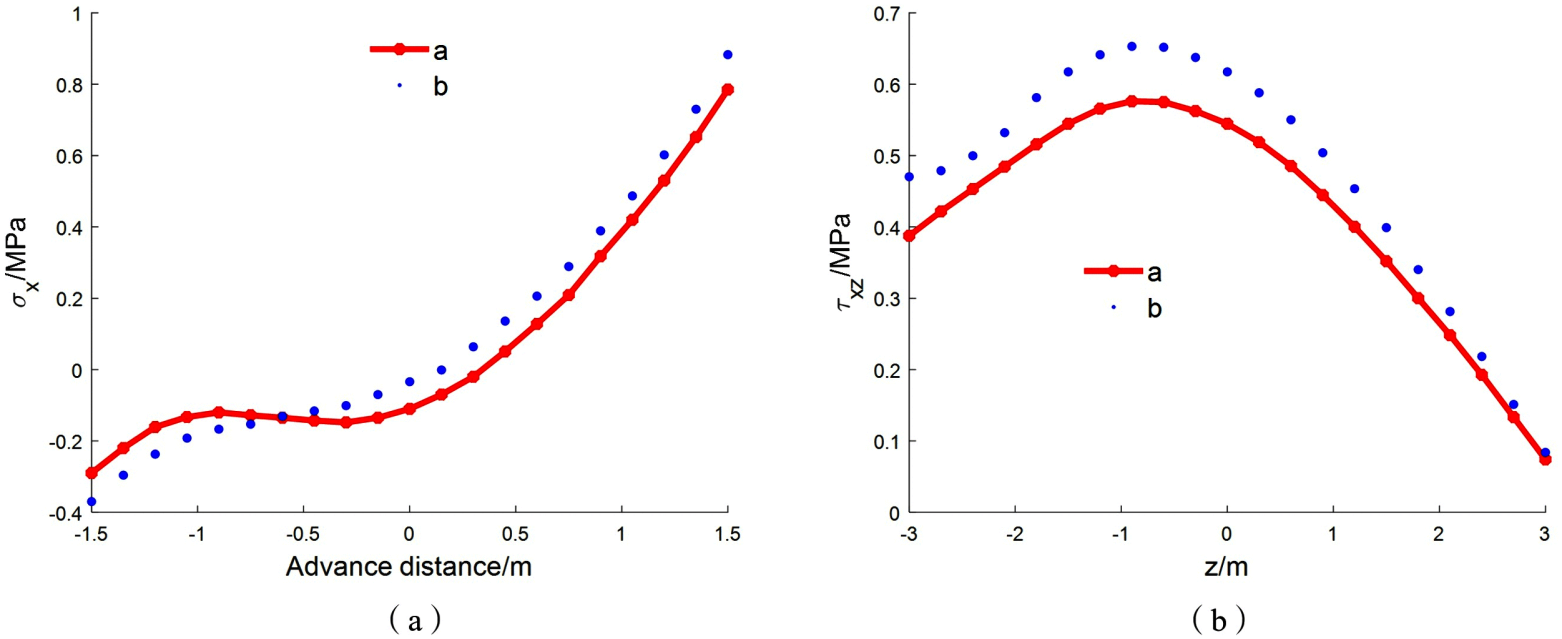
Stress comparison of the fault coal pillar at an aspect ratio of *h/L* = 3: (a) Comparison of mid-span normal stress in the key stratum; (b) Shear stress comparison.

**Fig 11:**
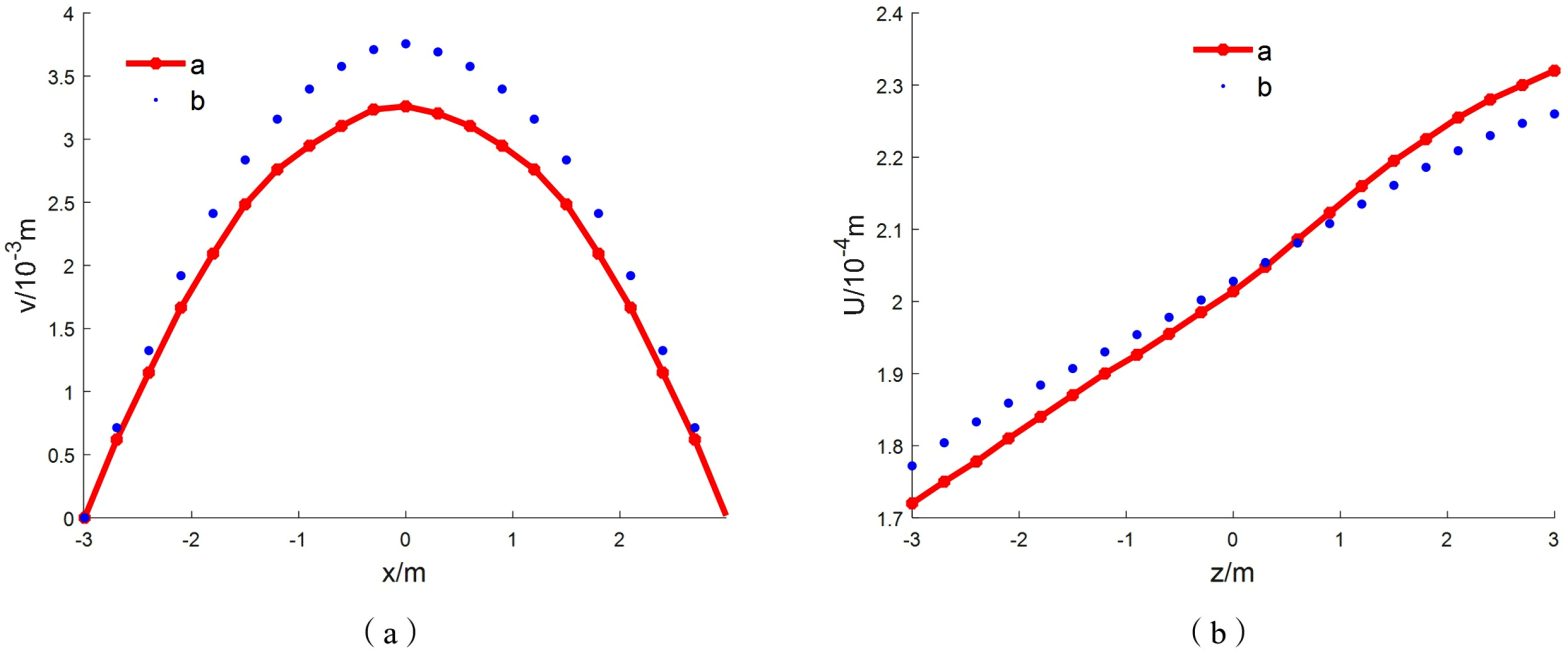
Displacement comparison of the fault coal pillar at an aspect ratio of *h/L* = 3: (a) Variation of *v* with *x* at *z* = 0m; (b) Variation of *u* with *z* at *x* = 1 m.

As shown in Figs 10 and 11, when the retained coal pillar width is set to 6 m—corresponding to a coal pillar width of 6 m and an aspect ratio of *h*/*L* = 3 with a layer thickness of 0.375 m—the maximum tensile stress in the pillar calculated by the layered deep-beam method is 0.785 MPa. At the mid-span position *x* = 1 m, the maximum horizontal displacement along the *z*-axis is 0.0238 cm for the analytical solution (solution a) and 0.0223 cm for the numerical solution (solution b), yielding a relative error of 6.3%. Along the *x*-axis, the maximum vertical displacements for solutions a and b are 0.342 cm and 0.375 cm, respectively, with a relative error of 8.81%.In the upper one-third section of the model, specifically within the range x = 0 to 0.6 m, the variation of shear stress along the *z*-axis shows that the maximum shear stress obtained from the analytical solution (solution a) and the numerical solution (solution b) are 0.576 MPa and 0.653 MPa, respectively, corresponding to a relative error of 11.1%.

According to the maximum tensile stress calculated using the layered deep-beam theory, it is found that when the aspect ratio reaches 3, the maximum tensile stress within the coal pillar is lower than the coal’s tensile strength. Therefore, the minimum required width of the fault water-resisting coal pillar is 6 m.

For comparison with conventional design methods, the empirical formula commonly used for calculating the required coal pillar width includes a safety factor. When the safety factor is removed for comparative purposes, the formula yields a required width of 1.93 m, which is significantly smaller than the result obtained using the layered deep-beam decomposition method. This discrepancy arises because conventional material mechanics-based formulas do not account for interlayer compressive stress, resulting in an underestimation of the maximum tensile stress—consistent with earlier conclusions in this study. When a safety factor is applied, the result (approximately 5.81 m) aligns closely with the theoretical value obtained from the layered deep-beam model, highlighting the essential role of the safety factor in the rational design of fault water-resisting coal pillars.

It is worth noting that the determination of coal pillar width in fault zones is a complex problem. In practical engineering applications, a lower limit of 20 m is often adopted. However, the focus of this study is not to determine the final design width, but rather to demonstrate the applicability of the layered deep-beam decomposition method in modeling the internal stress distribution of deep coal pillars. The results indicate that, compared to conventional formulas, the proposed method offers higher accuracy in stress prediction. This validates the effectiveness of the decomposition approach in simulating deep-beam behavior and provides theoretical and engineering guidance for the prevention of fault-induced water hazards in coal mines.

## 4. Conclusion

1. Stresses and displacements in the key stratum under uniformly distributed hydraulic pressure were solved using both the elasticity-based stress-function approach and the layered deep-beam decomposition method. Compared with FLAC3D simulations, the layered method provides consistently closer predictions, supporting the validity of the proposed framework.
2. As the aspect ratio increases, the layered deep-beam decomposition method captures the downward migration of the neutral axis in good agreement with numerical results, whereas the classical elasticity-based solution fixes the neutral axis near the section mid-height and becomes unsuitable for deep-beam conditions.
3. Increasing the number of layers in the decomposition improves the computational accuracy; however, the improvement exhibits a diminishing return. Therefore, in engineering practice, the degree of layer refinement in deep-buried rock beam models should be reasonably controlled. Additionally, as the aspect ratio increases, the calculation error of the layered deep-beam decomposition method also tends to grow, indicating that excessively wide layers may introduce significant deviations and should be avoided.
4. Considering the characteristics of fault water-resisting coal pillars—where the coal seam thickness is generally smaller than the pillar width—the problem can be regarded as a typical deep-beam scenario. The proposed layered deep-beam decomposition method was applied to determine a reasonable pillar width and was compared with results from the conventional empirical formula. The comparison indicates that the conventional material-mechanics-based formula tends to underestimate the maximum tensile stress under deep-beam conditions because it is derived from slender-beam assumptions and does not explicitly account for through-thickness compression and shear transfer. Consequently, the pillar width predicted without a safety factor is smaller than that obtained from the deep-beam-based analysis. Importantly, once a safety factor is introduced, the empirical prediction becomes consistent with the theoretical value from the layered method. This finding suggests that the safety factor used in practice effectively compensates for the model-form deficiency (and associated uncertainties) inherent in conventional formulations, and the proposed framework provides a clearer mechanical rationale for its engineering necessity in fault-adjacent water-resisting coal pillar design.

## Supporting information

S1 Table Source data for Figs 5, 9, 10 and 11. (XLSX)
